# Interpretación de la curación periapical en imágenes radiológicas. Una revisión

**DOI:** 10.21142/2523-2754-0904-2021-087

**Published:** 2021-12-09

**Authors:** Eloísa Archila-Montañez, Paola Elena Medina-Ocampo

**Affiliations:** 1 Especialista en Endodoncia de la Fundación Universitaria San Martín. Bogotá, Colombia. seam.od@hotmail.com Especialista en Endodoncia Fundación Universitaria San Martín Bogotá Colombia seam.od@hotmail.com; 2 Universidad Científica del Sur. Lima, Perú. paomedinaocampo@gmail.com Universidad Científica del Sur Universidad Científica del Sur Lima Peru paomedinaocampo@gmail.com

**Keywords:** radiolucidez periapical, integridad de la lámina dura, espacio para el ligamento periodontal, densidad del hueso periapical, imagen diagnóstica, curación periapical, periapical radiolucency, lamina dura integrity, periodontal ligament space, periapical bone density, diagnostic imaging, periapical healing

## Abstract

La interpretación mediante imágenes radiológicas del estado de curación de una periodontitis apical crónica o de un curetaje apical con apicectomía, se basa en el análisis de los cambios en el aspecto del área periapical, en la estructura del hueso adyacente al sitio de la inflamación o de la intervención quirúrgica, que se observan proyectados sobre estructuras óseas normales como las corticales. Por esto, al interpretar radiografías, se deben considerar limitaciones propias como la distorsión de estructuras o la superposición de imágenes anatómicas. En los estudios tradicionales de evaluación del área periapical después de un tratamiento, apoyados en imágenes 2D, los casos considerados exitosos o los fallidos se han comparado con un estándar de referencia, como el índice periapical o los criterios de evaluación del resultado del tratamiento quirúrgico endodóntico. Actualmente, algunos autores han referido otras condiciones que podrían intervenir en la evaluación, por ejemplo, que la apariencia radiográfica de la corona y la raíz del diente a evaluar pueden influir en la interpretación del área periapical. Adicionalmente, la tomografia computarizada de haz cónico (TCHC) permite relacionar el tejido lesionado o en proceso de reparación con las tablas vestibular y lingual para medir su periferia en una reconstrucción transversal. En efecto, durante el proceso de curación de los tejidos periapicales y radiculares, se presentan eventos biológicos complejos y mecanismos que pueden ocurrir a un mismo o en diferente tiempo. El conocimiento de la diversidad de tejidos es fundamental para identificar la dinámica de los signos de regeneración o si se produjo la curación por reparación.

Por tales razones, el propósito de este artículo fue identificar en la literatura actual los criterios utilizados para interpretar la curación periapical en imágenes bidimensionales o tridimensionales, y su relación con el diagnóstico y tratamiento.

## Abstract (en)

Interpretation of the state of healing of chronic apical periodontitis or apical curettage with apicoectomy in radiological images is based on the analysis of changes in the appearance of the periapical area and in the bone structure adjacent to the site of inflammation or surgical intervention, which are projected onto normal bone structures. However, distortion of structures or the superposition of anatomical images should be considered when interpreting radiographs. We compared conventional studies of evaluation of the periapical area following treatment, together with 2D images, with the periapical index and the criteria of evaluation in both successful and unsuccessful cases of endodontic surgical treatment. Some studies have reported that the radiographic appearance of the tooth crown and root may facilitate interpretation of the periapical area. In addition, cone-beam computed tomography can show the relationship between injured and repairing tissue using buccal and lingual tables to measure the periphery in transverse reconstruction. Indeed, complex biological events and mechanisms can occur during the healing process of periapical and root tissues. Knowledge of the diversity of tissues is essential to identify the dynamics of the signs of regeneration or the presence of healing due to repair. Hence, the aim of this article was to identify the criteria used to interpret periapical healing in two-dimensional or three-dimensional images and their relationship with diagnosis and treatment according to the literature currently available.

## INTRODUCCIÓN

La evaluación radiográfica de las lesiones periapicales resulta de gran interés en endodoncia, especialmente para la valoración de los resultados del tratamiento o como información en estudios clínicos epidemiológicos ^1^. Dado que una de las finalidades del tratamiento de conductos es prevenir o tratar la periodontitis periapical, es fundamental un examen radiológico preciso que permita detectarla o evaluar sus características y extensión, para planificar el tratamiento ^(2^^,^^3^.

**Figura 1:**
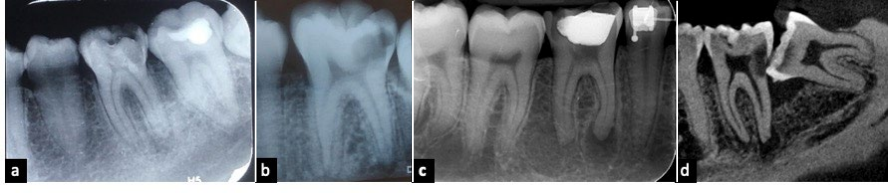
a) Radiografía periapical, diente 36; b) Radiografía periapical, diente 36; c) Radiografía periapical, diente 46; d) Imagen de CT, en corte sagital de diente 37. Se evidencian cambios en el aspecto de los tejidos periapicales de dientes con caries extensa o recurrente a restauración.

El término periodontitis apical (PA) se usa para determinar el proceso de la inflamación periapical que, por lo regular, resulta de la contaminación por microbios provenientes de la pulpa dental. Estos, junto con sus toxinas, traspasan la barrera de la lesión periapical y los mecanismos de defensa del organismo fracasan en limitar la injuria; entonces, la lesión se vuelve crónica, ante lo cual la respuesta del huésped es una inflamación persistente, que tratará de limitar la dispersión de la infección. Estos eventos ocurren en detrimento de los tejidos periapicales, lo que origina una lesión osteolítica visible en imágenes radiológicas ^4^^,^^5^ (Figura 1).

Sin embargo, la apariencia de una lesión puede cambiar de acuerdo con el momento en que se encuentre el proceso crónico, que es dinámico, debido a que el balance entre el agente infeccioso y la respuesta del organismo puede variar y provocar que la lesión se reagudice de manera secundaria, con la consecuente aparición de uno o más de los signos clínicos cardinales de la inflamación ^4^^,^^6^. 

Estas etapas de desarrollo y también de curación de la periodontitis apical crónica se manifiestan en cambios en el aspecto radiográfico del área periapical, en la estructura del hueso adyacente al sitio de la inflamación, sobre un fondo de estructuras óseas normales como las corticales ^6^. La interpretación debe considerar que las radiografías periapicales y panorámicas presentan limitaciones propias como la distorsión de estructuras o la superposición de imágenes anatómicas sobre las raíces de los dientes, lo cual puede generar “ruido anatómico” ^4^^,^^7^.

En los estudios clásicos de evaluación radiográfica del área periapical después de un tratamiento, tanto los casos considerados exitosos como los fallidos se han comparado con un estándar de referencia, tal como el índice periapical o los criterios de evaluación del resultado del tratamiento después de realizar cirugía endodóntica ^2^^,^^8^^-^^10^. Sin embargo, una investigación clínica basada en la posible influencia de la apariencia radiográfica intrarradicular y coronal sobre la interpretación de las áreas periapicales plantea preocupaciones respecto a las evaluaciones utilizadas como medida de resultados durante décadas ^11^. 

Actualmente, la tomografía computarizada de haz cónico (TCHC) permite detectar lesiones de origen endodóntico antes de que se hagan evidentes en radiografías convencionales, y relacionarlas con las tablas vestibular y lingual para medir la periferia de la lesión osteolítica en una reconstrucción transversal ^10^^,^^12^. Un estudio del año 2008 propuso un nuevo índice periapical, interpretando la radiolucidez periapical en escáneres de tomografía de haz cónico (TCHC) en 3 dimensiones: bucopalatina, mesodistal y diagonal ^13^.

En efecto, durante el proceso de curación de los tejidos periapicales y radiculares se presentan eventos biológicos complejos y mecanismos que pueden ocurrir a un mismo o en diferente tiempo. El conocimiento de los diferentes tejidos es fundamental para identificar signos de regeneración o si se produjo curación por reparación ^14^. Por tales razones, el propósito de este artículo de revisión fue identificar en la literatura actual los criterios imagenológicos utilizados para interpretar la curación periapical, en imágenes bidimensionales o tridimensionales, y su relación con el diagnóstico y tratamiento.

## MATERIALES Y MÉTODOS

En la búsqueda y selección de artículos para esta revisión, se siguieron los siguientes pasos: para empezar, se consultó en el vocabulario estructurado y multilingüe DeCS (descriptores en ciencias de la salud), la disponibilidad de los términos a utilizar en la búsqueda y recuperación de artículos científicos disponibles en las fuentes de información Medline, LILACS y otras, disponibles en la Biblioteca Virtual en Salud (BVS). Se consultaron los términos: “*periapical tissue*” y “*periapical periodontitis*”; luego, estos términos se consultaron por separado en la base de datos MeSH (*medical subjet headings*), que es el diccionario de vocabulario de términos médicos controlado por la National Library of Medicine, el cual se utiliza para indexar artículos para Pubmed. Además, se adicionaron los subtítulos “*diagnostic imaging*” y “*pathology*” con la operación lógica “*and/or*”. En el caso de “*periapical Tissue*”, se hallaron 484 resultados, los cuales se filtraron con una antigüedad no mayor a 5 años y se obtuvo 11 resultados. Para las palabras claves “*periapical periodontitis*” se encontraron 1120 resultados, a los que se aplicaron los filtros de una antigüedad no mayor a 5 años y relacionados con el tema, y se obtuvo 51 resultados.

Entre estos resultados se escogieron los artículos que contienen uno o más de los siguientes tópicos: curación apical, evaluación de lesión periapical, aspectos radiológicos o imagenológicos de lesiones apicales, regeneración apical, resultado de tratamiento convencional de conductos o de tratamiento quirúrgico apical. La selección final fue de 29 artículos para esta revisión.

### Características imagenológicas de normalidad en los tejidos perirradiculares

El conocimiento de los tejidos comprometidos en una lesión o en una herida quirúrgica es fundamental para formular el pronóstico del caso. Con respecto a la región periapical, el periodonto de inserción se encuentra conformado por cemento, ligamento periodontal y hueso alveolar derivados del ectomesénquima cefálico, específicamente del saco dental ^15^^,^^16^.

El cemento es un tejido conjuntivo mineralizado que se deriva de la capa celular ectomesenquimal del saco o folículo que rodea al germen dentario. Recubre la dentina radicular y su función primordial es anclar a la raíz del diente las fibras del ligamento periodontal. Pero, adicionalmente, al depositarse durante toda la vida, controla el ancho del espacio periodontal, equilibra el desgaste del diente en caso de atrición, lleva a cabo la reparación de la superficie de la raíz y transfiere las fuerzas oclusales al ligamento periodontal ^15^^,^^16^. Radiográficamente, tiene una radiopacidad semejante a la del hueso compacto y menor que la de la dentina. 

El ligamento periodontal es una capa delgada de tejido conjuntivo fibroso denso que rodea los dientes y, a través de sus fibras, une el cemento radicular con la lámina dura del hueso alveolar propio. Su función esencial es asegurar el diente dentro del alvéolo, tolera las fuerzas masticatorias y actúa como receptor sensorial. Está compuesto por sustancia fundamental amorfa, una gran cantidad de vasos, nervios y células, entre las que predominan los fibroblastos, pero se incluyen células productoras de matriz orgánica de hueso o de cemento; células que reabsorben hueso o cemento; macrófagos y otras células del sistema inmunitario; restos epiteliales de Malassez; células del sistema nervioso y células pluripotentes de origen ectomesenquimal ^15^^,^^16^. 

**Figura 2:**
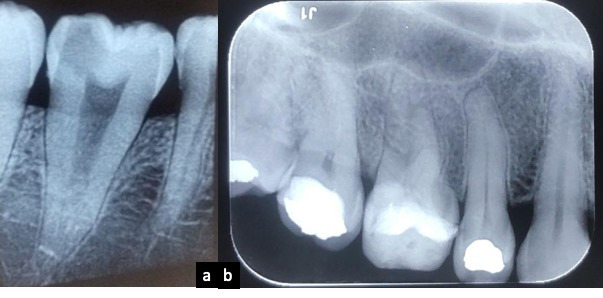
Radiografías periapicales: a) Diente 37; b) Diente 15. Se evidencia el espacio del ligamento periodontal, la lámina dura y la relación con el tejido pulpar.

Radiográficamente, el espacio fisiológico para el ligamento periodontal se observa como un espacio radiolúcido delgado entre la lámina dura y la raíz del diente, dentro del cual está inmerso el ligamento periodontal, que a nivel de la corona se correlaciona con el corion gingival y en la zona apical tiene relación con el tejido pulpar. El espacio para el ligamento periodontal tiene forma de reloj de arena, más angosto en el tercio medio de la raíz. El espesor del ligamento difiere considerablemente de acuerdo con la edad, la zona del diente y la función; se ha descrito comúnmente entre 0,10 y 0,38 µm o 0,2 y 0,4 mm (15) (Figura 2).

El hueso alveolar reviste el alvéolo; su función principal es facilitar que las fuerzas generadas por los movimientos masticatorios se distribuyan y sean absorbidas por la apófisis alveolar. El cumplimiento de esta función implica que el hueso alveolar, para compensar los movimientos dentarios, se renueve permanentemente gracias a que las células contenidas en él tienen la capacidad de fabricar, mantener y remodelar al tejido óseo ^16^.

**Figura 3:**
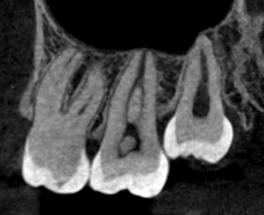
Imagen de CT, en corte sagital, dientes 26, 27 y 28. Se evidencia el proceso alveolar y la disposición de las trabéculas óseas al igual que la lámina dura que cubre internamente el alvéolo.

En imágenes diagnósticas, se puede observar el proceso alveolar que rodea los dientes, la apófisis alveolar, con un aspecto de red, de densidad variable, ya que están formados por hueso esponjoso, de origen medular que se caracteriza por trabéculas más gruesas y dispuestas en forma regular en el maxilar inferior y delgadas e irregulares en el maxilar superior. El hueso alveolar propiamente dicho forma parte del periodonto de inserción, tiene origen en el saco o folículo dental, se forma con el diente y desaparece con él; es un hueso laminar que cubre internamente el alvéolo y radiográficamente se observa como una delgada línea radiopaca que se denomina lámina dura ^15^^,^^16^ (Figura 3).

### Estado del arte de la curación periapical en imágenes 2D

La etiología de la periodontitis apical puede ser una infección primaria persistente, una infección secundaria como resultado de un tratamiento endodóntico fallido, en el cual los irritantes permanecen dentro del conducto radicular, o una infección extrarradicular que no se puede erradicar mediante un tratamiento convencional del conducto; además, se puede originar en una fractura vertical de la raíz ^4^^,^^17^.

La cicatrización depende del tipo de tejido lesionado o de la clase de herida quirúrgica ocasionada, por esto puede ser diversa y compleja. La cronología y los sucesos que tienen lugar durante la curación periapical es poco conocida; se presume que, desde la capa interna que reviste el hueso y desde el ligamento periodontal, las células pluripotentes de origen ectomesenquimal se diferencian en osteoblastos para sintetizar hueso y cementoblastos, para sintetizar cemento; también pueden diferenciarse en fibroblastos ^18^. De igual manera, se sabe poco sobre las características radiográficas de una periodontitis apical en estado de curación debido la dinámica de los procesos en que se fundamenta la compleja regeneración, que además involucra a todos los tejidos ^6^. 

Algunos autores definen la cicatrización como un proceso de curación mediante tejido conjuntivo, fibroplasia o fibrosis, que ocupa el espacio causado por la lesión y hace posible que el tejido remanente siga en funcionamiento, aunque no se recupere por completo, y se denomina reparación. La regeneración se define como el reemplazo de las células dañadas por unas diferenciadas nuevas que cumplen a totalidad la misma función sin dejar rastro del daño inicial; pero esta capacidad no la posee la generalidad de los tejidos, por lo que no siempre ocurre ^18^^,^^19^.

**Figura 4:**
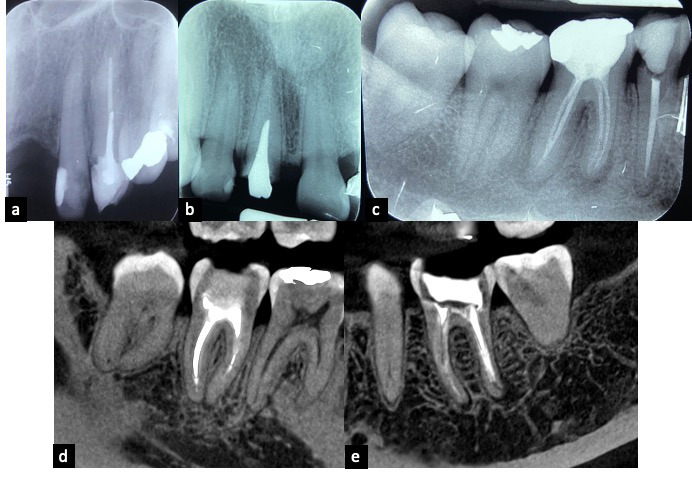
a) Radiografía periapical, diente 23; b) Radiografía periapical, diente 11; c) Radiografía periapical, diente 45 y 46; d) Imagen de CT, corte sagital de diente 47; e) Imagen de CT, corte sagital de diente 36. Dientes con conductos obturados asociados con algún tipo de cambio ósea estructural o rarefacciones que pueden estar relacionadas con procesos de curación prolongados.

En concordancia, después del tratamiento de conducto se puede presentar un aumento transitorio de la radiolucidez periapical que puede deberse a una irritación química o mecánica como secuela. Si hay un área con pérdida de minerales, gradualmente aumenta la densidad radiográfica llenándose de hueso, aunque la estructura de este hueso recién formado está menos organizada y puede diferir de la normal. Así, los contornos, el ancho y la estructura del ligamento periodontal y de la lámina dura vuelven a la normalidad. Si la cortical vestibular o lingual se encuentra perforada, la curación comienza desde el exterior hacia el interior de la lesión ^6^. La mayoría de dientes con conductos obturados se encuentran asociados a algún tipo de cambio óseo estructural o pequeña rarefacción, que puede estar relacionada con una fase de curación prolongada ^9^ (Figura 4). 

Es posible que el desarrollo de un tejido cicatricial dificulte el diagnóstico periapical, sobre todo si después de una cirugía endodóntica la curación ocurre por tejido cicatricial fibroso en lugar de hueso. Cuando se destruye una cantidad considerable de hueso y de periostio, se desarrolla un tejido fibroso de apariencia redonda, perforada y sin signos de irritación, el cual tiene un aspecto radiográfico típico y no necesita tratamiento adicional ^5^^,^^6^^,^^10^. Algunas características radiográficas que pueden ayudar a identificar este tipo de curación son la disminución de la rarefacción con respecto a la lesión inicial, con contornos irregulares que se extiende hacia el espacio periodontal, que además puede o no tener estructuras óseas internas visibles y se observa la lámina dura alrededor del ápice separando la rarefacción del diente ^5^^,^^20^. El tiempo que demora la curación también es poco conocido; algunos estudios de seguimiento han realizado controles a 1 año y otros hasta 7 años o más después del tratamiento ^20^^-^^23^. 

### Índice periapical como criterio de interpretación del estado periapical

El índice periapical (IP) se ha utilizado como sistema de puntuación para la evaluación radiográfica de la periodontitis apical, pues ofrece una escala de referencia visual y asigna un estado de salud a la raíz. Con base en radiografías de referencia y diagnóstico histológico verificado, su puntuación tiene en cuenta la apariencia radiográfica del periodonto normal, los cambios en la estructura del hueso periapical por alguna pérdida de mineral y la desmineralización del hueso periapical con área radiolúcida. Lo clasifica en diferentes grupos, entre ellos estructuras periapicales normales o periodonto apical normal; pequeños cambios en la estructura del hueso periapical o cambios estructurales sugestivos, pero no patognomónicos de enfermedad periodontal; cambios en la estructura del hueso periapical o en el hueso estructural con algún grado de pérdida de mineral característica de periodontitis apical; desmineralización del hueso periapical con área radiolúcida bien definida o radiolucidez bien definida; y desmineralización del hueso periapical con características de exacerbación o radiolucidez en forma de expansiones radiadas del hueso estructural ^9^^,^^12^.

### Curación después de cirugía endodóntica como criterio de interpretación del estado periapical

La cicatrización posterior a una cirugía endodóntica depende de varios factores; por esto, el concepto debe sustentarse en la evaluación clínica y radiográfica de la región periapical. Sin embargo, dado que la evaluación clínica es limitada y el examen radiográfico se supone que es el reflejo de los cambios histológicos de la región periapical, la evaluación que aporta mejor información sobre la condición periapical es la evaluación histológica, pero no es un método que se pueda usar siempre ^14^^,^^21^^,^^25^.

Andreasen y Rud ^20^ trataron de evaluar en su estudio la conexión entre los hallazgos radiográficos y el estado histológico periapical. Posteriormente, se proponen por primera vez unos criterios radiográficos que requieren observación de por lo menos un año después de haber realizado cirugía apical, y describen la lámina dura y el espacio del ligamento periodontal alrededor del ápice, al igual que la apariencia del hueso adyacente. De acuerdo con el seguimiento, si estas estructuras se volvieron a formar completa o parcialmente, si hubo variación en ancho del espacio del ligamento periodontal comparado con el de zonas del diente no afectadas por la lesión y la presencia de rarefacciones en la densidad radiográfica del hueso peri radicular, clasificaron esta curación en cuatro grupos de ocurrencia frecuente.

### Interpretación del estado periapical con técnicas 3D

**Figura 5:**
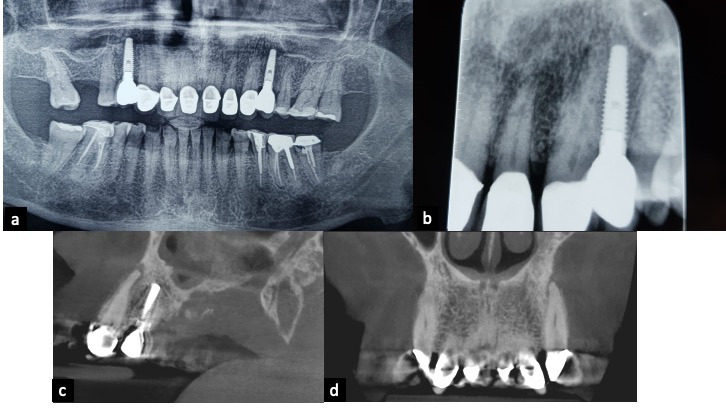
a) Radiografía panorámica; b) Radiografía periapical, diente 23; c) Imagen de CT, corte sagital, diente 23; d) Imagen de CT, corte coronal, diente 23. Imágenes diagnósticas influyen en la decisión de tratamiento.

Las técnicas radiológicas permiten ver los cambios en la mineralización y en la estructura del hueso periapical, que son los principales indicadores de la presencia de periodontitis apical y de la progresión de la curación. Por tanto, la información diagnóstica influye directamente sobre las decisiones de tratamiento ^12^ (Figura 5). 

**Figura 6:**
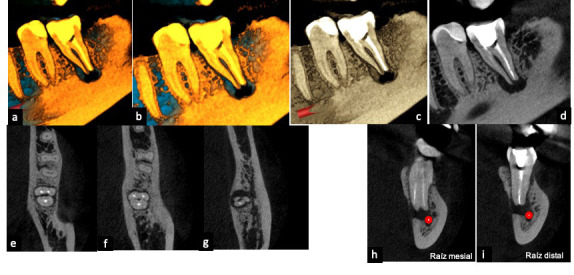
Imágenes de CT: a) y b) Renderizado 3D; c) y d) Corte sagital; e), f) y g) Cortes axiales; h) e i) Cortes coronales. Cortes de tomografía cone-beam de diente 37, para seguimiento de los resultados del tratamiento.

Se requieren imágenes avanzadas que sean eficaces y, entre otros propósitos, permitan evidenciar las coordenadas anatómicas de las lesiones en las tres dimensiones espaciales con fines de diagnóstico y tratamiento, para determinar las medidas y forma de la lesión, la cantidad de cortical ósea involucrada y visualizar la relación con el ápice de la raíz y los puntos anatómicos óseos. Estas técnicas también facilitan diferenciar lesiones originadas en la pulpa dental (granuloma o quiste) y lesiones de origen endodóntico frente a otras lesiones de aspecto radiolúcido. Y, no menos importante, permiten realizar evaluación de seguimiento de los resultados del tratamiento ^12^^,^^24^^,^^26^^-^^29^ (Figura 6).

## DISCUSIÓN

En presencia de una periodontitis apical, los mecanismos de defensa del organismo se activan para tratar de limitar la dispersión de la infección ^5^; sin embargo, puede ocurrir que no logren controlar el daño, ya sea porque el agente causal no se ha retirado, porque el tratamiento convencional de conductos o incluso el quirúrgico apical no logró los resultados esperados, o debido a factores implícitamente relacionados con la curación de los tejidos injuriados. 

Una vez instaurada la periodontitis apical, la particularidad de condición crónica implica que ocurran eventos propios de la inflamación y de la respuesta huésped, todos ellos en perjuicio de los tejidos periapicales, lo que origina una lesión osteolítica visible en imágenes radiológicas. Por lo tanto, la apariencia de los tejidos periapicales en una imagen diagnóstica puede cambiar de acuerdo con el momento dinámico en que se encuentre un proceso crónico; también cambia si este llega a sufrir una agudización o si, por el contrario, exhibe signos de restablecimiento de la normalidad o algún tipo de curación ^6^.

El propósito de esta revisión fue explorar en la literatura actual los criterios para la interpretación de la curación periapical en imágenes.

La mayor cantidad de artículos se relacionan con periodontitis apical observada en imágenes 2D y, en menor número, en imágenes 3D. Gran parte de ellos se enfocan en el éxito o fracaso del tratamiento convencional o quirúrgico de la periodontitis apical, o en la relación entre el material de obturación del conducto o de obturación retrógrada en apicectomía y los resultados del tratamiento. Algunos hacen la correlación de las lesiones extraídas con estudios histopatológicos y casi todos contrastan el resultado con el tiempo de control a 3 meses o más después del tratamiento. Estos diferentes enfoques se consideran una limitante, ya que no hay consenso sobre criterios para evaluar la curación apical en sí misma.

En concordancia, esta revisión permitió clarificar conceptos anatómicos y funcionales de los tejidos periapicales y su relación con la apariencia imagenológica. Así como el periodonto de inserción se encuentra conformado por cemento, ligamento periodontal y hueso alveolar; el cemento es el tejido que ancla a la raíz del diente las fibras del ligamento periodontal. Adicionalmente, al depositarse durante toda la vida, controla el ancho del espacio periodontal, equilibra el desgaste del diente en caso de atrición y lleva a cabo la reparación de la superficie de la raíz, entre otras funciones.

El ligamento periodontal rodea los dientes y, a través de sus fibras, une el cemento radicular con la lámina dura del hueso alveolar propio. La lámina dura reviste el alvéolo y cumple la función de facilitar que las fuerzas generadas por los movimientos masticatorios se distribuyan y sean absorbidas por la apófisis alveolar. Debido a eso, se renueva permanentemente gracias a que las células contenidas en el ligamento periodontal tienen la capacidad de fabricar, mantener y remodelar al tejido óseo ^16^.

Con respecto a la cicatrización, algunos autores la definen como un proceso de curación mediante tejido conjuntivo, fibroplasia o fibrosis, que ocupa el espacio causado por la lesión y hace posible que el tejido remanente siga en funcionamiento, aunque no se recupere por completo y se denomina reparación. En tanto, la regeneración se define como el reemplazo de las células dañadas por unas diferenciadas nuevas que cumplen a totalidad la misma función sin dejar rastro del daño inicial; pero esta capacidad no la posee la generalidad de los tejidos, por lo que no siempre ocurre ^19^.

Es posible que el desarrollo de un tejido cicatricial dificulte el diagnóstico periapical en imágenes, sobre todo si después de una cirugía endodóntica la curación ocurre por formación de tejido cicatricial fibroso en lugar de hueso; dado el caso, al destruirse una cantidad considerable de hueso y de periostio, se desarrolla un tejido fibroso de apariencia redonda, perforada y sin signos de irritación, el cual tiene un aspecto radiográfico típico y no necesita tratamiento adicional.

Para la evaluación radiográfica de la periodontitis apical, se ha utilizado un sistema de puntuación: el índice periapical (IP), que ofrece una escala de referencia visual y asigna un estado de salud a la raíz con base en radiografías de referencia y diagnóstico histológico verificado, su puntuación tiene en cuenta la apariencia radiográfica del periodonto normal, los cambios en la estructura del hueso periapical por alguna pérdida de mineral y la desmineralización del hueso periapical con área radiolúcida ^9^.

Andreasen y Rud ^21^ trataron de evaluar en su estudio la conexión entre los hallazgos radiográficos y el estado histológico periapical. Posteriormente, se proponen, por primera vez, unos criterios radiográficos apreciables después de haber realizado cirugía apical como tratamiento a periodontitis periapical, que requieren observación de por lo menos un año y describen la lámina dura y el espacio del ligamento periodontal alrededor del ápice, al igual que la apariencia del hueso adyacente. De acuerdo con el seguimiento, si estas estructuras se volvieron a formar completa o parcialmente, si hubo variación en el ancho del espacio del ligamento periodontal, comparado con el de zonas del diente no afectadas por la lesión y en virtud de la presencia de rarefacciones en la densidad radiográfica del hueso perirradicular, clasificaron esta curación en cuatro grupos de ocurrencia frecuente ^10^.

En contraste, los estudios relacionados con los resultados de tratar la periodontitis periapical con un tratamiento convencional de conductos no han producido un consenso sobre los criterios de evaluación de la curación periapical.

## CONCLUSIONES

Las técnicas radiológicas permiten ver los cambios en la mineralización y en la estructura del hueso periapical, principales indicadores de la presencia de periodontitis apical; por lo tanto, se requieren más estudios específicos sobre las características imagenológicas en 2D y 3D de la curación apical, que permitan llegar a un consenso en su diagnóstico.

## Fuente de financiamiento:

Autofinanciado

## References

[1] Camps J, Pommel L, Bukiet F (2004). Evaluation of periapical lesion healing by correction of gray values. J Endod.

[2] Kruse C, Spin-Neto R, Christiansen R, Wenzel A, Kirkevang LL (2016). Periapical bone healing after apicectomy with and without retrograde root filling with mineral trioxide aggregate A 6-year follow-up of a randomized controlled trial. J Endod.

[3] Alghamdi F, Alhaddad AJ, Abuzinadah S (2020). Healing of periapical lesions after surgical endodontic retreatment a systematic review. Cureus.

[4] Huamán-Chipana P, Cortés-Sylvester MF, Hernández M (2015). Evaluación de lesiones periapicales de origen endodóntico mediante tomografía computada Cone Beam. Ciencias Clínicas.

[5] Karamifar K, Tondari A, Saghiri MA (2020). Endodontic periapical lesion an overview on the etiology, diagnosis and current treatment modalities. Eur Endod J.

[6] Huumonen S, Orstavik D (2002). Radiological aspects of apical periodontitis. Endod Top.

[7] Leonardi Dutra K, Haas L, Porporatti AL, Flores-Mir C, Nascimento Santos J, Mezzomo LA (2016). Diagnostic accuracy of cone-beam computed tomography and conventional radiography on apical periodontitis A systematic review and meta-analysis. J Endod.

[8] Kruse C, Spin-Neto R, Reibel J, Wenzel A, Kirkevang LL (2017). Diagnostic validity of periapical radiography and cBct for assessing periapical lesions that persist after endodontic surgery. Dentomaxillofacial Radiol.

[9] Kerekes K, Hm E, Orstavik D, Kerekes K (1986). Dental Traumatology.

[10] Molven O, Halse A, Grung B (1987). Observer strategy and the radiographic classification of healing after endodontic surgery. Int J Oral Maxillofac Surg.

[11] Strong JW, Woodmansey KF, Khademi JA, Hatton JF (2017). Coronal and intraradicular appearances affect radiographic perception of the periapical region. J Endod.

[12] Cotti E (2010). Advanced techniques for detecting lesions in bone. Dent Clin North Am.

[13] Estrela C, Bueno MR, Azevedo BC, Azevedo JR, Pécora JD (2008). A new periapical index based on cone beam computed tomography. J Endod.

[14] Alvarado Masó A (2003). Cicatrización de los procedimientos quirúrgicos en endodoncia.

[15] Lindhe J, Lang N, Karring T (2009). Periodontologia clinica e implantologia odontologica.

[16] Gómez de Ferraris CM (2002). Histología y embriología bucodental.

[17] Liao WC, Lee YL, Tsai YL, Lin HJ, Chang MC, Chang SF (2019). Outcome assessment of apical surgery A study of 234 teeth. J Formos Med Assoc.

[18] Kumar V, Abbas AK, Fausto N, Aster JC (2015). Robbins y Cotran.Patología Estructural y Funcional.

[19] Garrett S (1996). Periodontal regeneration around natural teeth. Ann Periodontol.

[20] Andreasen JO, Moller Jensen JE (1972). Radiographic criteria for the assessment of healing after endodontic surgery. Int J Oral Surg.

[21] Andreasen JO, Rud J (1972). Correlation between histology and radiography in the assessment of healing after endodontic surgery. Int J Oral Surg.

[22] Molven O, Halse A, Grung B (1996). Incomplete healing (scar tissue) after periapical surgery - Radiographic findings 8 to 12 years after treatment. J Endod.

[23] Tassoker M, Akgunlu F (2016). Radiographic evaluation of periapical status and frequency of endodontic treatment in a turkish population a retrospective study. J Istanbul Univ Fac Dent.

[24] Tassoker M, Akgunlu F (2016). Radiographic evaluation of periapical status and frequency of endodontic treatment in a turkish population a retrospective study. J Istanb Univ Fac Dent.

[25] Hirsch JM, Ahlstrom U, Henrikson PA, Heyden G, Peterson LE (1979). Periapical surgery. Int J Oral Surg.

[26] Yan MT (2006). The management of periapical lesions in endodontically treated teeth. Aust Endod J.

[27] Ogutlu F, Karaca I (2018). Clinical and radiographic outcomes of apical surgery a clinical study. J Maxillofac Oral Surg.

[28] Tyndall DA, Rathore S (2008). Cone-Beam CT diagnostic applications caries, periodontal bone assessment, and endodontic applications. Dent Clin North Am.

[29] Juerchott A, Pfefferle T, Flechtenmacher C, Mente J, Bendszus M, Heiland S (2018). Differentiation of periapical granulomas and cysts by using dental MRI a pilot study. Int J Oral Sci.

